# Femoral Head and Liner Exchange in Patients with Atraumatic Dislocation. Results of a Retrospective Study with 6 Years Follow-Up

**DOI:** 10.3390/medicina57111188

**Published:** 2021-11-01

**Authors:** Maximilian G. Hanslmeier, Michael W. Maier, Manuel Feisst, Nicholas A. Beckmann

**Affiliations:** 1 Department of Orthopaedics and Trauma Surgery, Heidelberg University Hospital, Schlierbacher Landstr 200A, 69118 Heidelberg, Germany; Maximilian.Hanslmeier@med.uni-heidelberg.de; 2 Swabian Joint Center, Christoph Straße 7, 70178 Stuttgart, Germany; maier@sgz-stuttgart.de; 3 Institute of Medical Biometrics and Informatics, Heidelberg University Hospital, Im Neuenheimer Feld 130.3, 69120 Heidelberg, Germany; feisst@imbi.uni-heidelberg.de

**Keywords:** arthroplasty, hip, THA, dislocation, liner

## Abstract

*Background and Objectives*: Femoral head and liner exchange is an established treatment for polyethylene wear but has had a more limited role in the treatment of other conditions including dislocation, because of concerns about an increased postoperative dislocation rate. Some authors have considered dislocation associated with polyethylene wear to be a contraindication for this procedure. *Materials and Methods*: Our retrospective review evaluated the outcome of head and liner exchange in a small consecutively operated heterogeneous cohort of 20 patients who presented with dislocation unrelated to trauma, component malposition or component loosening. Of this group, 12 had prior primary total hip arthroplasty, and 8 had prior revision total hip arthroplasty, and included 4 patients with prior revision for dislocation. Mean follow-up was 6 ± 3.5 years (range 1–145 months). *Results*: Kaplan–Meier analysis revealed a revision-free implant survival from any cause of 80% (confidence interval 95%:64.3–99.6%) at 5 years after head and liner exchange (index surgery). At final follow-up, 83.3% of patients (*n* = 10) with prior primary total hip arthroplasty and 62.5% of patients (*n* = 5) with prior revision total hip arthroplasty, had not required subsequent revision for any cause. None (0%) of the primary total hip arthroplasty group and 3 (38%) of the revision arthroplasty group had required revision for further dislocation. Of the eight revision arthroplasty patients, four had a prior revision for dislocation and three of these four patients required further revision for dislocation after index surgery. The fourth patient had no dislocation after index surgery. One additional patient who had prior revision surgery for femoral component fracture suffered dislocation after index surgery, but was successfully treated with closed reduction. *Conclusions*: In our study population, femoral head and liner exchange was an effective treatment option for patients with prior primary total hip arthroplasty and also for a highly select group of revision total arthroplasty patients with no prior history of dislocation. Femoral head and liner exchange does not appear to be a viable treatment option for patients who have had revision total arthroplasty after prior dislocations.

## 1. Introduction

Total hip arthroplasty (THA) is one of the most successful orthopedic procedures with 10-year survival rates of 90% and an annually increasing frequency in a progressively younger patient population [1]. However, a minority of patients suffer severe consequences with joint instability, the most common postoperative morbidity, and the second most frequent cause of revision surgery after component loosening [2,3,4].

A dislocation rate of 0.3% to 10% has been reported after Primary THA (PTHA) [5,6,7] and up to 28% after Revision THA (RTHA) [2,6].

The majority of dislocations are single episodes (2/3) and can be treated conservatively with closed reduction. Surgical intervention is frequently required for recurrent and late episodes [3,8,9], and several surgical strategies are utilized. Revision THA is most commonly utilized with a variety of components including constrained or lipped liners and dual mobility (DM) cups. Removal of a well-fixed acetabular cup results in bone loss [3].

Less frequently, less invasive modular component exchange is utilized in patients with a well-fixed acetabular component and a well-fixed femoral stem, adequately functioning abductor mechanism and absence of component malposition [3,8,10,11]. Head and liner exchange is a shorter, less complex procedure than RTHA involving fixed component exchange and conserves bone stock [3]. Prior studies of modular component exchange for the treatment of dislocation are few and have shown variable results. One prior study included PTHA cases only. Study cohorts varied from 11–48 hips with follow-up periods of 36–69 months [3,8,9,12,13]. Re-dislocation rate varied from 0% [9] to 55% [3].

Our current study of 20 patients treated for dislocation with modular component exchange differs from prior similar studies by the longer follow up period and a more diverse patient population that included patients with prior RTHA for recurrent dislocation.

The goal of our study was to assess the effectiveness of head and liner exchange for the treatment of dislocation in our PTHA and RTHA groups and our cohort as a whole. We hypothesized that patients with prior revision for dislocation would have a higher dislocation rate after index surgery.

## 2. Materials and Methods

Between January 2019 and September 2019 we utilized our internal database and conducted a retrospective chart review of 94 consecutively operated hips (92 patients) who received femoral head and liner exchange (index surgery) for a variety of causes between January 2004 and December 2013, at our hospital. Of this group, 34 hips (33 patients) were treated for periprosthetic infection, 23 hips (22 patients) were treated for polyethylene wear, 17 hips (17 patients) were treated for various causes (metallosis, inlay dislocation, ceramic head fracture) and 20 hips (20 patients) were treated for dislocation and are the focus of this study.

Exclusion criteria included evidence of component malposition or loosening as identified by clinical and/or radiographic examination, dislocation resulting from trauma and combination surgical procedures. Our study was approved by the local University Ethics Committee (Ethikkommission der Medizinischen Fakultaet Heidelberg, File Number S-548/2016).

### 2.1. Patient Demographics and History

Our cohort consisted of 20 patients (20 hips), 10 men and 10 women, who presented with dislocation. No patient was lost to follow up, but one patient died from unrelated causes during the study period at 126 months post index surgery and is included in the study.

At index surgery, patient mean age was 63 ± 13 years (range 27–79) and the mean BMI was 29.2 ± 6.9 kg/m^2^ (range 20.1–44.6). The right hip was revised in 8 patients and the left hip in 12. All patients were classified according to the American Society of Anesthesiologists (ASA) classification [14]; Class I: 2 patients, Class II: 9 patients, Class III: 9 patients. Table 1 and Table 2 display the diagnoses that necessitated prior hip procedures for both patient groups (PTHA and RTHA).

In 12 patients the index surgery was the first revision surgery after PTHA (Table 1), and 8 patients had at least 1 prior revision procedure for a variety of causes including prior revisions for dislocation in 4 RTHA patients (Table 2).

The mean time interval between the index procedure and the prior procedure was 62.4 ± 51.4 months (range 0.75–170 months) for the PTHA group and 26.6 ± 26.3 months (range 2–72 months) for the RTHA group.

### 2.2. Preoperative Assessment

Pre-operative assessment included clinical assessment, routine labs, and AP and axial (Lauenstein) radiographs with the patient in the supine position for assessment of component position [15]. Acetabular cups and femoral stems were well fixed radiographically [16,17]. All cups were well positioned and the inclination and anteversion angles were in the Lewinnek ‘safe zone’ [18]. The mean inclination angle was 41 ± 6.4 degrees, and the mean anteversion angle was 16 ± 6.1 degrees (measurement done using AP radiographs and TraumaCAD^®^ software (TraumaCAD^®^, Brainlab AG, Munich, Germany). These were confirmed intraoperatively. CT scans were used to evaluate cases of questionable malposition or loosening. CT scans allow more accurate assessment of component malposition and are considered the gold standard [19]. Scintigraphy with Tc was not used.

### 2.3. Surgical Procedure and Follow Up

The surgical approach utilized the prior approach to minimize scarring and soft tissue deficiency. It was transgluteal in 11 patients, anterolateral in 5 and non-standard (modified transgluteal or anterolateral) in 4 RTHA patients with multiple prior surgeries and preexisting osseous and soft tissue deficiencies (Girdlestone procedure and proximal femoral fracture) (Table 2).

All index procedures were performed by surgeons experienced in hip surgery; however, consecutive surgeries were not always done by the same surgeon. Intra-operatively stable acetabular cup fixation was confirmed, and component malposition was excluded. The liner locking mechanisms was examined and confirmed to be intact in all cases.

The femoral head size was increased in 13 hips, decreased in 1 hip and unchanged in 2 hips. The pre-operative head size was not documented in 4 hips, but all received a 32 mm or larger replacement femoral head (Table 1 and Table 2). All patients received peri-operative intravenous cefuroxime and postoperative thrombo-embolic prophylaxis.

After modular exchange, mobility and stability of the joint was tested intra-operatively by taking the operated hip through a complete range of motion (ROM). Impingement was excluded by confirming clearance between the greater trochanter and the pelvis and between the metal femoral neck and polyethylene or metal rim, with the limb positioned in maximal extension and external rotation and in 90 degrees flexion and maximal internal rotation [20].

Patients were followed in the outpatient clinic at 3 and 6 months post index procedure and yearly thereafter for a mean of 6.0 ± 3.5 years or until dislocation, demise or revision surgery.

We looked at 3 study endpoints:

1. Incidence of postoperative dislocation.
2. Implant survival with no further surgical intervention for dislocation; overall and for PTHA and RTHA groups separately.
3. Implant survival from all causes.

### 2.4. Statistical Analysis

Patient characteristics were described using descriptive statistics. The re-revision free survival was described using Kaplan–Meier estimators; the 60-month survival rate of the implants for all causes was described using corresponding 95% confidence intervals (CI).

The software SPSS^®^ Version 22.0 (SPSS Inc, Chicago, IL, U.S.A.), SAS^®^ Version 9.4 (SAS Institute Inc., Cary, NC, USA) and Microsoft Excel (Microsoft, Redmond, WA, USA) were used to record and analyze the data.

## 3. Results

**Figure 1:**
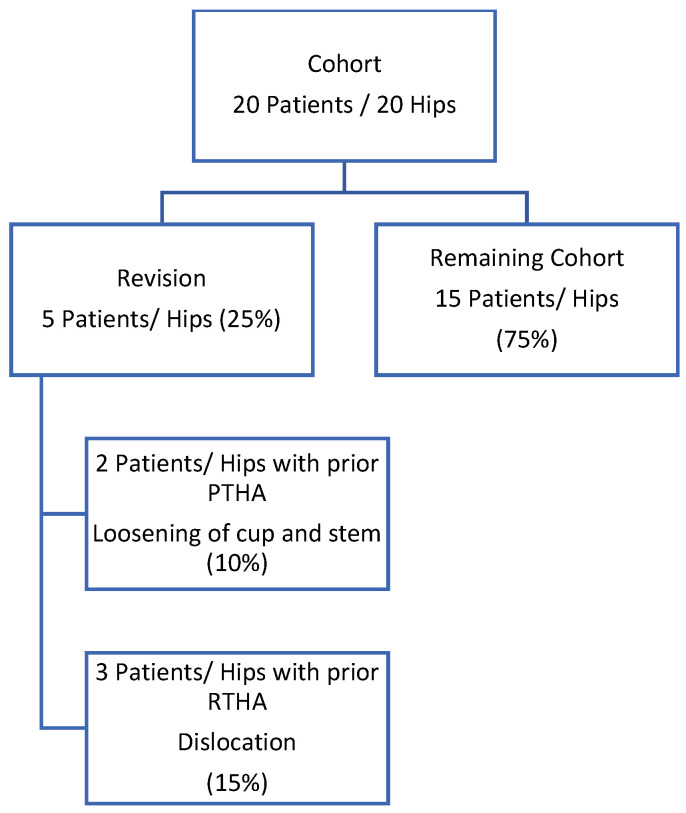
Flow chart of the patient cohort.

An overview of patient outcome is represented in the flow chart (see Figure 1).

Twenty patients (20 hips) received femoral head and liner exchange for treatment of dislocation. One patient died of unrelated causes and with no dislocation at 126 months post index surgery, and is considered a treatment success. Twelve patients (hips) had prior PTHA and eight patients had prior RTHA (Table 1 and Table 2).

**Figure 2:**
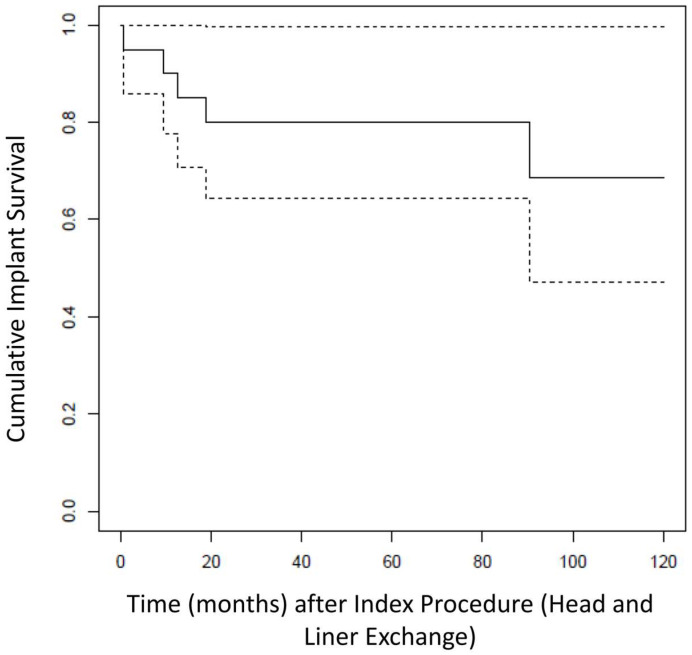
Kaplan–Meier analysis showing the overall re-revision free survival (solid line) from all causes of the THAs after head and liner exchange for dislocation (dotted lines represent upper and lower CI).

Kaplan–Meier Analysis revealed a re-revision-free implant survival from all causes of 80.0% (CI95%: 64.3–99.6%) at 5 years after head and liner exchange (see Figure 2). At final follow up of 6.0 ± 3.5 years, five patients required surgical re-revision for any cause (25%). Implant survival from any cause was 75% (15 patients).

Of the five patients who required re-revision, further dislocation was the cause in three patients; each had a history of RTHA and prior surgical treatment for dislocation. Implant survival from further dislocation was 85% (17 patients) (Table 2). The two remaining patients who required re-revision experienced component loosening (one femoral stem and one acetabular component). Both were PTHA patients (Table 1).

One additional RTHA patient with a history of femoral component fracture, also experienced post index surgery dislocation, but this was successfully treated with closed reduction (Table 2).

The incidence of dislocation post-index surgery was four hips (20%).

## 4. Discussion

Our results are comparable to prior studies despite our heterogeneous patient population including some at very high risk. At mean 6 years follow up, we achieved 100% and 50% success (no dislocation) in our PTHA and RTHA groups respectively. Lachiewicz et al. reported 82% and 50% success in their PTHA and RTHA groups respectively with small cohorts and shorter follow up periods [12]. Biviji et al. defined success as up to one postoperative dislocation and achieved 76% and 64% success in their PTHA and RTHA groups respectively [8]. Our overall implant survival with no additional surgery for dislocation was 17/20 (85%), postoperative dislocation was 4/20 (20%), and implant survival from all causes was 15/20 (75%). Our four patients who developed hip dislocation had multiple prior procedures with re-revision for dislocation in three patients and included the only patient who received a constrained liner during index surgery. Re-revision for aseptic loosening was required in two additional patients; one femoral shaft and one acetabular cup.

Hip instability is the most frequent cause of morbidity after THA [3] and up to 70% of dislocations occur in the first 3–6 months post THA [2,4]. Approximately 2/3 of first dislocations can be successfully treated conservatively [8]. Up to 42% of dislocations are recurrent and require surgical intervention [21,22]; dislocation is the most common reason for early revision THA [2]. Trabecular Metal™ (TM) shells have been associated with a high rate of postoperative dislocation [7,23].

Charissoux et al. defined three categories based on time of presentation from index surgery: 3–6 months resulting from inadequate healing; up to 5 years related to resumed activities; more than 5 years resulting from polyethylene wear [2]. Component malposition and trauma also contribute to late occurrence [9,24].

Revision surgery for instability has had variable success and is directed at cause if identified [25]. Preoperative history, physical exam, lab and radiologic studies with CT scan if necessary are important to identify periprosthetic infection, component malposition, and P/E wear that influence subsequent surgical procedures. Interventions for dislocation include revision of a malpositioned component, trochanteric advancement, increase of femoral head size, implantation of bipolar and tripolar cups or constrained acetabular components and femoral head and liner exchange [2,3,8,9,11,12,13,26,27]. Combined procedures are sometimes employed [28].

Femoral head and liner exchange is a treatment option in cases with a stable well implanted acetabular cup that is not malpositioned [3,8,10] and has been utilized increasingly in cases with P/E wear [29] with reports of implant survival that are comparable to those of more complex procedures [30]. Head and liner exchange is considered controversial by some when used for other indications [30] and has had limited use in the treatment of periprosthetic joint infection, femoral head and liner dissociation, liner fracture and/or detachment and instability of the hip [11]. Published literature on the topic of femoral head and liner exchange for treatment of dislocation following THA is relatively sparse.

Advantages of femoral head and liner exchange over RTHA with exchange of well-fixed components for the treatment of dislocation include the avoidance of significant bone loss associated with the removal of a well-fixed component, a shorter operative and recovery time, and decreased blood loss [29]. These advantages must be weighed against the disadvantages of possible incomplete debridement of osteolytic areas and granulomas [29] and an increased postoperative dislocation rate as reported in some studies [3,30,31,32]. Retention of a cup with a damaged or inferior locking mechanism can be managed by cementation of a liner [11]. Concerns about an increased postoperative dislocation rate have resulted in the relatively infrequent use of head and liner exchange in cases other than P/E wear [11], and in some studies the presence of associated dislocation was an exclusion criterion [26,29,30,32,33].

Possible risk factors for dislocation are patient, procedure and component related. Guo et al. conducted a systematic review and meta- analysis of risk factors after RTHA and found that a history of instability and prior revisions was the most significant risk factor as a result of bone loss, soft tissue damage and abductor insufficiency [34]. An increasing number of prior revisions was associated with an increasing risk of dislocation [4]. Cumulative rates of dislocation increase over time, with an increase of 1% every 5 years after the first year [35] with a 35% cumulative re-dislocation rate 15 years after revision for dislocation [36]. Our results are consistent with these findings; our cohort included six patients with two prior procedures, one with three prior procedures, and one with four prior procedures, placing them at high risk for subsequent dislocation. Of these, four patients had a prior history of hip dislocation and three of these suffered re-dislocation and required re-revision. The fourth was successfully treated with closed reduction (Table 2).

Other patient risk factors are neuromuscular and cognitive impairments that decrease ability to co-operate postoperatively (i.e. Parkinson’s disease, alcoholism, dementia) [4,6,27], advanced age associated with diminished tissue healing [35,37] and BMI >30 kg/m^2^ [4,38]. Our patient with BMI 43 kg/m^2^ suffered dislocation but was successfully treated with closed reduction. Patients with lumbosacral pathology including sagittal spine deformity or prior spine surgery have abnormal pelvic tilt that influences acetabular cup position and are at greater risk of postoperative dislocation [39,40].

Acetabular component malposition has been identified as a possible cause of recurrent instability in up to 1/3 of cases [19,41]. Lewinnek et al. found that acetabular cup orientation significantly influenced dislocation after THA and proposed a ‘safe zone’ for cup inclination and anteversion angles after PTHA of 40 ± 10 and 15 ± 10 degrees respectively, measured radiographically with the patient supine [18]. Lewinnek’s safe zone is not applicable when standing due to spino-pelvic tilt [42]. However, some studies have documented >55% of dislocated cups were within the safe zone [25,43]. Our cohort had mean cup inclination and anteversion angles within the safe zone as measured radiographically and intraoperatively.

A posterior surgical approach has been considered a risk factor, since the majority of dislocations occur posteriorly [4,27,44,45,46]. Kwon et al. in their meta-analysis found that this was only true with inadequate or no capsular repair [45]. The majority (11) of our patients had a transgluteal approach, five had antero-lateral and four had non-standard approaches as a result of extensive muscular and osseous defects [45].

Choice of components can influence outcome. Femoral head diameter, neck length and head neck ratio affect dislocation risk [47]. Larger heads have greater ROM and ‘jump’ distance with less chance of impingement; 22 mm heads are associated with more risk than larger heads [2,6,26,35] and 28 mm heads have greater risk than 32 mm [10,34]. Howie et al. found that 28 mm heads had 5× more dislocations than 36 mm. Liner type also affects risk and is reduced with elevated rim liners [26,48]. Constrained components have been associated with inadequate locking mechanisms and increased cup loosening with poor results in some studies [3,10,49,50]. More recently dual mobility (DM) cups have received increasing attention and usage in RTHA including cases of dislocation, with results superior to those of constrained liners [51]. Lange et al. [52] reported a 5% recurrent dislocation rate after first time revision for instability. De Martino et al. in their systemic review found a 3% dislocation rate in RTHA in high risk patients [53]. Bruggemann reported a 1.4% dislocation rate after cementing dual mobility cups into TM shells (off-label use) [7]. However, there is rarely intraprosthetic dislocation, a unique complication, and possible accelerated P/E wear with resultant osteolysis, prompting caution for use in younger patients [53,54].

This retrospective review has a number of limitations including the possibility of incomplete documentation in some cases and lack of a control group. Our patient cohort was small and prohibited the assessment of variables that could influence the results that are not statistically significant because of the small number. Procedures were done by several surgeons and variations in technique cannot be excluded, although all followed the hospital protocol.

Comparison with other studies is difficult because of differences in patient cohorts, definitions of failure, components and surgical techniques utilized and follow up periods.

Although our study has a small patient cohort, it serves as an addition to a sparse body of information that relates to femoral head and liner exchange in the treatment of dislocation.

## 5. Conclusions

The advantages of our protocol compared to RTHA involving exchange of well-fixed components include shorter operative time, less complex surgery and preservation of bone stock.

Our cohort consisted of 20 patients, 12 with prior PTHA and eight with prior RTHA. All 12 patients with prior PTHA had no history of prior surgery for dislocation and were effectively treated with femoral head and liner exchange.

The eight patients with prior RTHA included four with prior RTHA for dislocation; of these four patients, three suffered a further dislocation after head and liner exchange and required further revision. One additional patient with a history of revision for femoral component fracture, experienced dislocation after head and liner exchange and was successfully treated with closed reduction.

For the entire cohort, implant survival from dislocation was 85% and implant survival from all causes was 75% at a mean follow up of 6 ± 3.5 (range 0.08–11.8) years. Two PTHA patients required later revision for component loosening.

Our results suggest that for patients with prior PTHA and no prior history of dislocation, this protocol could be a viable treatment option. However, patients with a history of multiple prior THA’s that include treatment for instability are at high risk of further dislocation.

The literature on femoral head and liner exchange in dislocation cases is sparse to date, therefore additional studies would be of great value.

## Figures and Tables

**Table 1:** Head and liner exchange for dislocation after primary THA. Abbreviations: OA = osteoarthritis, THA = total hip arthroplasty, HXLPE = highly crosslinked polyethylene, UHMWPE = ultrahigh molecular weight polyethylene, MoM = metal on metal articulation).

Primary Indication	Primary Operation	Primary Head Size (mm)	Gender	Age at Head + Liner Exchange	Index Surgery (Revision Head Size (mm))	Inlay	Follow up (Months)	Complications
OA	THA	28	M	50	32	HXLPE lipped	126	-
OA	THA	32	M	62	36	HXLPE	65	-
OA	THA	28	M	66	28	UHMWPE	145	
OA	THA	28	F	71	36	HXLPE	93	
Dysplasia OA	THA	unknown	F	58	32	UHMWPE lipped	104	-
OA	THA	unknown	F	62	32	UHMWPE	9	Loosening femoral stem
OA	THA	MoM	M	71	32	HXLPE lipped	54	-
Dysplasia OA	THA	28	M	38	32	UHMWPE lipped	90	Cup loosening
OA	THA	MoM	F	79	36	HXLPE	57	-
Femoral neck fracture	THA	28	F	76	32	UHMWPE lipped	66	-
OA	THA	28	M	69	36	HXLPE	114	-
OA	THA	28	M	21	36	HXLPE	75	-

**Table 2:** Head and liner exchange for dislocation after RTHA (Abbreviations: OA = osteoarthritis, THA = total hip arthroplasty, HXLPE = highly crosslinked polyethylene, UHMWPE = ultrahigh molecular weight polyethylene, CRIF = closed reduction internal fixation).

Prior to Index Procedure (Head and Liner Exchange)				Index Procedure (Head and Liner Exchange)				
Indication for Arthroplasty	Surgery Performed	Prior Head Size (mm)	Gender	Age at Head + Liner Exchange	New Head Size (mm)	Liner	Follow-Up (Months)	Complications
OA	(1) Primary THA (2) Head and liner exchange for metallosis							
		28	F	58	28	HXLPE lipped	142	-
OA	(1) Primary THA (2) Shaft, head, liner exchange for shaft loosening							
		28	F	75	32	HXLPE Lipped	50	-
OA	(1) Primary THA (2) Shaft, head, liner exchange for dislocation							
		28	F	59	32	UHMWPE	73	-
OA	(1) Primary THA (2) THA revision for aseptic loosening of a thrust plate prosthesis							
		32	F	62	36	HXLPE	76	-
OA secondary to dysplasia	(1) Primary THA (2) Cup revision with head and liner exchange for dislocation							
		28	M	61	36	HXLPE Lipped	12	Dislocation, Re-revision
OA	(1) Primary THA (2) Explantation and Girdlestone for PJI (3) Revision THA with re-implantation (4) Head and Liner exchange for dislocation							
						
						
		32	M	73	28	HXLPE Constrained	18	Dislocation, Re-revision
OA secondary to acromegaly	(1) Primary THA (2) Shaft, Head, Liner Exchange for femoral component fracture							
		32	M	65	36	HXLPE	61	Dislocation, Closed reduction
Inter-trochanteric fracture	(1) CRIF with Gamma Nail (2) Conversion to THA (3) Head and Liner exchange for dislocation							
						
		28	F	77	32	HXLPE	1	Dislocation, Re-revision

## Data Availability

The data that support the findings of this study are available on request from the corresponding author.
